# In Vitro Characterization, Modelling, and Antioxidant Properties of Polyphenon-60 from Green Tea in Eudragit S100-2 Chitosan Microspheres

**DOI:** 10.3390/nu12040967

**Published:** 2020-03-31

**Authors:** Eliana B. Souto, Raquel da Ana, Selma B. Souto, Aleksandra Zielińska, Conrado Marques, Luciana N. Andrade, Olaf K. Horbańczuk, Atanas G. Atanasov, Massimo Lucarini, Alessandra Durazzo, Amélia M. Silva, Ettore Novellino, Antonello Santini, Patricia Severino

**Affiliations:** 1 Department of Pharmaceutical Technology, Faculty of Pharmacy, University of Coimbra, Pólo das Ciências da Saúde, Azinhaga de Santa Comba, 3000-548 Coimbra, Portugal; quele.ana@gmail.com (R.d.A.); zielinska-aleksandra@wp.pl (A.Z.); 2 CEB—Centre of Biological Engineering, University of Minho, Campus de Gualtar, 4710-057 Braga, Portugal; 3 Department of Endocrinology, Hospital de São João, Alameda Prof. Hernâni Monteiro, 4200-319 Porto, Portugal; sbsouto.md@gmail.com; 4 Laboratory of Nanotechnology and Nanomedicine (LNMED), Institute of Technology and Research (ITP), Av. Murilo Dantas, 300, Aracaju 49010-390, Brazil; conrado.marques@souunit.com.br (C.M.); pattypharma@gmail.com (P.S.); 5 Industrial Biotechnology Program, University of Tiradentes (UNIT), Av. Murilo Dantas 300, Aracaju 49032-490, Brazil; 6 Tiradentes Institute, 150 Mt Vernon St, Dorchester, MA 02125, USA; 7 Department of Physiology, Federal University of Sergipe, CEP São Cristóvão 49100-000, Sergipe, Brazil; luciana.nalone@hotmail.com; 8 Department of Technique and Food Product Development, Warsaw University of Life Sciences (WULS-SGGW) 159c Nowoursynowska, 02-776 Warsaw, Poland; olaf_horbanczuk@sggw.pl; 9 The Institute of Genetics and Animal Breeding, Polish Academy of Sciences, Jastrzębiec, 05-552 Magdalenka, Poland; atanas.atanasov@univie.ac.at; 10 Institute of Neurobiology, Bulgarian Academy of Sciences, 23 Acad. G. Bonchev str., 1113 Sofia, Bulgaria; 11 Department of Pharmacognosy, University of Vienna, 1090 Vienna, Austria; 12 Ludwig Boltzmann Institute for Digital Health and Patient Safety, Medical University of Vienna, Spitalgasse 23, 1090 Vienna, Austria; 13 CREA-Research Centre for Food and Nutrition, Via Ardeatina 546, 00178 Rome, Italy; massimo.lucarini@crea.gov.it (M.L.); alessandra.durazzo@crea.gov.it (A.D.); 14 Department of Biology and Environment, University of Trás-os-Montes e Alto Douro (UTAD), Quinta de Prados, 5001-801 Vila Real, Portugal; amsilva@utad.pt; 15 Centre for Research and Technology of Agro-Environmental and Biological Sciences (CITAB), University of Trás-os-Montes e Alto Douro (UTAD), 5001-801 Vila Real, Portugal; 16 Department of Pharmacy, University of Napoli Federico II, 80131 Napoli, Italy

**Keywords:** green tea, epigallocatechin gallate, chitosan, microspheres, Eudragit, metabolic diseases

## Abstract

Eudragit S100-coated chitosan microspheres (S100Ch) are proposed as a new oral delivery system for green tea polyphenon-60 (PP60). PP60 is a mixture of polyphenolic compounds, known for its active role in decreasing oxidative stress and metabolic risk factors involved in diabetes and in other chronic diseases. Chitosan-PP60 microspheres prepared by an emulsion cross-linking method were coated with Eudragit S100 to ensure the release of PP60 in the terminal ileum. Different core–coat ratios of Eudragit and chitosan were tested. Optimized chitosan microspheres were obtained with a chitosan:PP60 ratio of 8:1 (Ch-PP60_8:1_), rotation speed of 1500 rpm, and surfactant concentration of 1.0% (*m*/*v*) achieving a mean size of 7.16 µm. Their coating with the enteric polymer (S100Ch-PP60) increased the mean size significantly (51.4 µm). The in vitro modified-release of PP60 from S100Ch-PP60 was confirmed in simulated gastrointestinal conditions. Mathematical fitting models were used to characterize the release mechanism showing that both Ch-PP60_8:1_ and S100Ch-PP60 fitted the Korsmeyers–Peppas model. The antioxidant activity of PP60 was kept in glutaraldehyde-crosslinked chitosan microspheres before and after their coating, showing an IC_50_ of 212.3 µg/mL and 154.4 µg/mL, respectively. The potential of chitosan microspheres for the delivery of catechins was illustrated, with limited risk of cytotoxicity as shown in Caco-2 cell lines using the 3-(4,5-dimethylthiazole-2-yl)-2,5-diphenyltetrazolium bromide (MTT) assay. The beneficial effects of green tea and its derivatives in the management of metabolic disorders can be exploited using mucoadhesive chitosan microspheres coated with enteric polymers for colonic delivery.

## 1. Introduction

Green tea is obtained from the fresh leaves of *Camellia sinensis,* a plant from the *Theaceae* family which has been used for centuries as a natural antioxidating beverage. Its polyphenolic constituents provide it with additional therapeutic benefits in the modulation of oxidative stress-induced cardiovascular diseases associated with diabetes, already confirmed in clinical trials [1,2,3,4,5,6]. Green tea extracts, rich in antioxidant polyphenols (e.g., epigallocatechin-3-gallate), have been reported for reducing lipid peroxidation, oxidized low-density lipoproteins (LDL), cholesterol levels, and anti-hypertensive effects, factors that are relevant to reduce cardio-metabolic disease risk [7]. A catechin extract from green tea, containing a mixture of the main active green tea polyphenols components, named polyphenon-60 [8], has recently drawn attention. Indeed, the role of green tea polyphenon-60 (PP60) in decreasing metabolic risk factors, oxidative stress, inflammation, and in the amelioration of cardiac apoptosis in experimentally induced diabetes has been described [9]. Among the naturally derived catechins (flavonoids composing the majority of soluble solids of green tea extracts), epigallocatechin gallate (EGCG) has already been proposed as active ingredient in polymeric nanoparticles for oral administration [10], and as lipid nanoparticles for ocular administration [11,12].

From a quick search using as keywords “epigallocatechin and nanoparticles” 376 publications appeared indexed in the Web of Science dated between 2000 and 2020, while “green tea and nanoparticles” resulted in the list of 780 publications. When associating epigallocatechin and dyslipidemia/dyslipidemia [13], only 33 works were listed as published over the last 20 years.

While the molecular mechanisms involved in the effect of catechins on the metabolism of lipids and sugars remains to be fully described, catechins are known to induce antioxidant enzymes, inhibit pro-oxidant enzymes and scavenge reactive oxygen species (ROS), and to chelate metals [14,15,16]. EGCG has been reported to improve insulin-resistance and metabolic profiles, as well as to reduce adipocyte area as a consequence of lipolytic action [17]. Anti-obesity effects, such as inhibition of fatty acid absorption and reduction in leptin levels were reported in a high-fat diet rat model combined with green tea extract administration [18]. Green tea and its polyphenols have been widely reported to exhibit positive effects against inflammation, cancer, aging, and others [11,19]. 

Casanova et al. [20] reviewed the effect of epigallocatechin gallate (EGCG) on oxidative stress and inflammation linked to the metabolic dysfunction of skeletal muscle in obesity and their underlying mechanisms and highlighted that in order to overcome the problem of EGCG instability and low bioavailability, future direction is the use of nanocarriers [20]. Chitosan is a biodegradable linear biopolyaminosaccharide obtained by alkaline deacetylation of chitin, with several advantages for oral drug delivery. It is a non-toxic, mucoadhesive natural polymer, with a high charge density, showing not only the capacity to improve dissolution of drugs, but also to improve the fat metabolism in the body [21,22]. Chitosan is being extensively used in the production of drug delivery systems (e.g., silica nanoparticles, microspheres) for oral administration [22,23,24,25,26]. The production of microspheres can be achieved by the electrostatic interaction between the biopolyaminosaccharide and the low molecular counterions such as polyphosphates, sulphates, and cross-linking with glutaraldehyde producing a gel [27,28,29]. 

To be effective in the management of metabolic diseases, the oral administration route is of preference due to higher convenience and higher patient compliance. Polyphenon-60 contains pure catechins, mainly EGCG, and has been selected for the present work as the active ingredient to be loaded into chitosan microspheres coated with Eudragit S-100 for delayed release in the gut. The choice of the oral route is primarily due to it being non-invasive and appropriate for self-administration, which increases the success of the therapeutic outcome. However, the hydrophilic environment of the gastrointestinal tract (GIT) compromises the absorption of many sensitive drugs [22].

The aim of this work was the development of a delayed release oral formulation for PP60. Chitosan microspheres produced by the ionic cross-linking method was been loaded with PP60 and further coated with methacrylic anionic copolymers (Eudragit S-100) to obtain an enteric dosage form, capable of releasing the active in the ileum with improved bioavailability aiming the prevention of metabolic diseases. 

## 2. Materials and Methods

### 2.1. Materials

Polyphenon-60 (PP60, yellow powder with a total cathecin content >60%), chitosan from shrimp shells (molecular weight 150 kDa, 95% deacetylated, low viscosity <200 mPa∙s), glutaraldehyde, ascorbic acid, and Span 80 (sorbitan monooleate) were purchased from Sigma-Aldrich (Saint Louis, Missouri, USA). Eudragit S-100 (poly(methacylic acid-co-methyl methacrylate) 1:2) was received as a kind gift from Evonik (São Paulo, Brazil). Liquid paraffin was obtained from VWR Chemicals (Lisbon, Portugal). All other reagents (glacial acetic acid, monobasic potassium phosphate, sodium dihydrogen phosphate, sodium hydroxide, toluene, petroleum ether, acetone, ethanol, and methanol) were obtained from Reagente-5 (Porto, Portugal). For cell culture, Dulbecco’s modified Eagle’s medium (DMEM), fetal bovine serum (FBS), Minimum Essential Medium Eagle’s (MEME), Trypsin-0.3% EDTA, phosphate buffered saline (PBS), L-glutamine, non-essential amino acids (NEAA), sodium dodecyl sulfate (SDS), dimethylformamide (DMF), 3-(4,5-dimethylthiazol-2-yl)-2,5-diphenyltetrazolium bromide (MTT), Triton^®^ X, and gentamicin were purchased from Sigma-Aldrich (Saint Louis, MO, USA). Caco-2 cell line was purchased from American Type Culture Collection (ATCC, Pensabio Biotecnologia, São Paulo, Brazil). The water used in all experiments was ultrapure, obtained from a MilliQ^®^ Plus, Millipore^®^ (Germany).

### 2.2. Production of Chitosan Microspheres

Chitosan microspheres were produced by emulsion cross-linking, at room temperature and following the method described by Jose et al. [26]. Briefly, PP60 was added to a solution of 2% (*m*/*v*) chitosan prepared in 1% (*w*/*v*) of glacial acetic acid aqueous solution. From the obtained solution, a volume of 3 mL was sampled and injected into 20 mL of oil phase of paraffin containing Span 80 with a syringe (No. 23) under mechanical stirring (Ultra-Turrax, T18, IKA, Staufen, Germany) for 30 min to form a w/o emulsion. A volume of 1.5 mL of toluene-saturated glutaraldehyde (8:1) was then added to the obtained emulsion, which was left to stabilize and to cross-link over a period of 5.5 hours. The obtained microspheres were centrifuged at 4000 rpm, the precipitate washed with petroleum ether and acetone and dried in a laboratory hot air oven (Binder Inc, Germany) at 50 °C. A total of 10 batches were produced by varying the processing parameters as shown in Table 1. For the first set of batches (Ch-PP60_2:1_, Ch-PP60_4:1_, Ch-PP60_8:1_, and Ch-PP60_10:1_), the rotational speed was kept constant at 1500 rpm and the concentration of Span 80 in liquid paraffin was kept at 1% (*m*/*v*), and varying the chitosan:PP60 ratios. For the second set of batches (Speed_10_, Speed_15_, and Speed_20_), the chitosan:PP60 ratio was maintained at 4:1, the concentration of Span 80 in liquid paraffin was kept at 1% (m/v), and the rotational speed varied from 1000–2000 rpm. For the third set of batches (S80_0.5_, S80_1.0_, and S80_1.5_), the chitosan:PP60 ratio was maintained at 4:1, the rotational speed at 1500 rpm, while the concentration of surfactant (Span 80) in liquid paraffin ranged from 0.5%–1.5% (*m*/*v*).

### 2.3. Eudragit S-100 Coating of PP60-Loaded Chitosan Microspheres

The coating of Ch-PP60 microspheres with Eudragit S-100 to obtain S100Ch-PP60 was done by emulsion-solvent evaporation technique, as described by Jose et al. [26]. Ch-PP60 microspheres were suspended in a 10% (*w*/*v*) of Eudragit S-100 in ethanol (2.5 mL) and then emulsified in light liquid paraffin (40 mL) containing 1.0% (*w*/*v*) Span 80. To form a stable emulsion, 2 mL of ethanol was added drop wise. The emulsion was kept for 3 h under mechanical stirrer (Ultra-Turrax, T18, IKA, Staufen, Germany) at 1000 rpm. The S100Ch-PP60 was collected, rinsed with petroleum ether, and dried in a laboratory hot air oven (Binder Inc., Germany) at 50 °C.

### 2.4. Particle Size Analysis

The particle size of Ch-PP60 (uncoated) and S100Ch-PP60 (coated) microspheres was measured in an optical Zeiss microscope (Oberkochen, Germany) fitted with a calibrated eyepiece micrometer under a magnification of 40×. The diameter of about 100 microspheres was measured randomly and the average size (*D_mean_*) determined using the Edmondson’s equation [26]:

(1) $$D_{mean}=\frac{\sum nd}{n}$$

where *n* is the number of counted microspheres and *d* is the mean size range.

### 2.5. Yield of Production, Loading Capacity, and Encapsulation Efficiency

The yield of production (YP%) was calculated based on the dry weight of microspheres, applying the following equation:

(2) $$YP\%=\frac{W_{m}}{W_{PP60}+W_{c}}\times 100$$

where *W_m_* is the mass of produced microspheres, and *W_PP60_* and *W_c_* are the mass of PP60 and chitosan, respectively, initially taken for the production of the microspheres. For the determination of the loading capacity (LC%) and encapsulation efficiency (EE%), 10 mg of microspheres were weighted and triturated in a mortar and pestle with 20 mL methanol. The mixture was kept overnight for the extraction of the active from chitosan. After filtration and proper dilution with methanol, the absorbance was read in a UV spectrophotometer Shimadzu UV-1601 (Shimadzu Italy, Cornaredo, Italy) at 280 nm against a calibration curve for the quantification of EGCG ($W_{\mathrm{EGCG}\;\mathrm{read}\;\lambda 280\mathrm{nm}}$) [30]. The LC% and EE% were calculated using the following equations:

(3) $$LC\%=\frac{W_{EGCG\;read\;\lambda 280nm}}{W_{m}}\times 100$$

(4) $$EE\%=\frac{W_{EGCG\;read\;\lambda 280nm}}{Wm}\times 100$$

### 2.6. In Vitro Release Assay

The in vitro release of PP60 from Ch-PP60 (uncoated) and S100Ch-PP60 (coated) microspheres was evaluated in simulated gastrointestinal (GI) conditions using the United States Pharmacopoeia (USP) rotating paddle dissolution apparatus at 100 rpm and at 37 ± 0.5 °C, as described by Jose et al. [26]. Accurately weighed mass of microspheres, equivalent to 30 mg of PP60, was added to 450 mL of dissolution medium and GI conditions simulated over time by modifying the pH at pre-determined time intervals. From 0–2 h, the pH was kept at 1.2 by adding HCl (0.1 N). From 2–4 hours, 1.7 g of KH_2_PO_4_ and 2.225 g of Na_2_HPO_4_^∙^2H_2_O were added to the medium and the pH adjusted to 4.5 with NaOH (1.0 M). From 4–12 h, NaOH (1.0 M) was added to adjust the pH to 7.4. Over the course of the assay, and at pre-determined time intervals up to 12 hours, a volume of 2 mL was withdrawn from the medium and replaced with fresh dissolution medium to ensure sink conditions over the entire experiment. Samples were analyzed by reading the absorbance in a spectrophotometer Shimadzu UV-1601 (Shimadzu Italy, Cornaredo, Italy) at 280 nm against a calibration curve for the quantification of EGCG [30]. The effect of the chitosan:PP60 ratio on the in vitro drug release was analyzed and the best ratio compared to the coated formulation. All measurements were done in triplicate. The in vitro drug release data of the coated S100Ch-PP60 formulation was fitted to four kinetic models i.e., zero order, first order, Higuchi, and Korsemeyer–Peppas models [25], selecting the most appropriate model based on the obtained *R^2^* values.

### 2.7. Antioxidant Activity

#### 2.7.1. DPPH Assay

The antioxidant activity of PP60 was measured when loaded into chitosan microspheres, and the effect of the enteric Eudragit S-100 coating (Ch-PP60 versus S100Ch-PP60) was compared. The assay evaluated the ability of the loaded PP60 to scavenge the stable DPPH^•^ radical [31]. Briefly, microspheres (Ch-PP60; S100Ch-PP60) were dissolved in 0.1 mM DPPH methanolic solution. Then, a volume of 20 µL of sample was placed in the microplate wells to which 200 µL DPPH methanolic solution (0.1 mM) was added. Methanol was used as negative control and butylated hydroxytoluene (BHT, 0–6 µg/mL) was used as the positive control. The microplates were incubated at 25 °C for 30 min, and then read at 517 nm in a multiplate reader (DTX 880 Multimode Detector, Beckman Coulter Inc.). The percentage of the antioxidant activity (AA (%)) was calculated from the recorded optical densities (OD), using the following equation:

(5) $$\mathrm{AA}(\%)=\frac{\mathrm{OD}\;\mathrm{of}\;\mathrm{negative}\;\mathrm{control}\;-\mathrm{OD}\;\mathrm{of}\;\mathrm{sample}}{\mathrm{OD}\;\mathrm{of}\;\mathrm{negative}\;\mathrm{control}}\;\times 100$$

The linear regression equation was obtained by plotting the concentration in the X-axis (μg/mL) against AA(%) in the Y-axis (% inhibition), from which the IC_50_ value could be calculated.

#### 2.7.2. In Vitro Caco-2 Cells Proliferation Assay

The MTT assay was used for the evaluation of the proliferative capacity of Caco-2 cells when treated with Ch-PP60 (uncoated) and S100Ch-PP60 (coated) microspheres [32]. Caco-2 cell lines were firstly seeded in 96-well microtiter plates (0.1×10^6^ cells/mL; 100 μL/well). After 24 h of incubation, serum DMEM was replaced with serum free DMEM. The next day, cells were treated with the microspheres. Solutions of Ch-PP60 and S100Ch-PP60 in dimethyl sulfoxide (DMSO 0.7%) at gradient concentrations (0.5, 2.5, 5, 10, and 15 µg/mL of microspheres) were prepared in serum free DMEM, added to each well and incubated for more 24 and 48 h at 37 °C in a 5% CO_2_ atmosphere. A solution of DMSO 1% was set as the negative control, whereas a doxorubicin solution (100 μg/mL) was set as the positive control. At the end of the incubation period, test solutions were removed. MTT solution (150 μL) at 0.5 mg/mL was added to each well and incubated in the dark for 4 h at 37 °C in a 5% CO_2_ atmosphere. The experiments were repeated three times, and quadruplicates were done for each condition in each assay. Cell viability was determined as the ability of viable cells to reduce the yellow dye MTT to the purple formazan. The obtained precipitate was dissolved in 150 μL DMSO and the absorbance was read at 595 nm using a multiplate reader (DTX 880 Multimode Detector, Beckman Coulter Inc.). The results were expressed as percentage of cell viability in relation to the negative control as follows:

(6) $$Cell\;viability\left[\%\right]=\left[\frac{Abs_{Test}}{Abs_{Negative\;Control}}\times 100\right]$$

### 2.8. Statistical Analysis

All measurements were performed in triplicate, and results expressed as the mean ± S.D. Statistical significance was established at *p* < 0.05 and was calculated using a one-way analysis of variance ANOVA followed by the Tukeys Test. Values of *p* < 0.05 were considered significant. All statistical analyses were carried out using the GraphPad program 5.0^®^ (Intuitive Software for Science, San Diego, CA, USA).

## 3. Results

The microspheres produced by emulsion cross-linking between chitosan and glutaraldehyde to load PP60 were yellowish because of the natural color of the active ingredient. To select the best combination of chitosan and PP60, and the production parameters, the particle size (*D_mean_*), yield of production (YP%), loading capacity (LC%), and encapsulation efficiency (EE%) were determined for the different batches (as shown in Table 1), and the results of the physicochemical characterization are given in Table 2.

**Figure 1:**
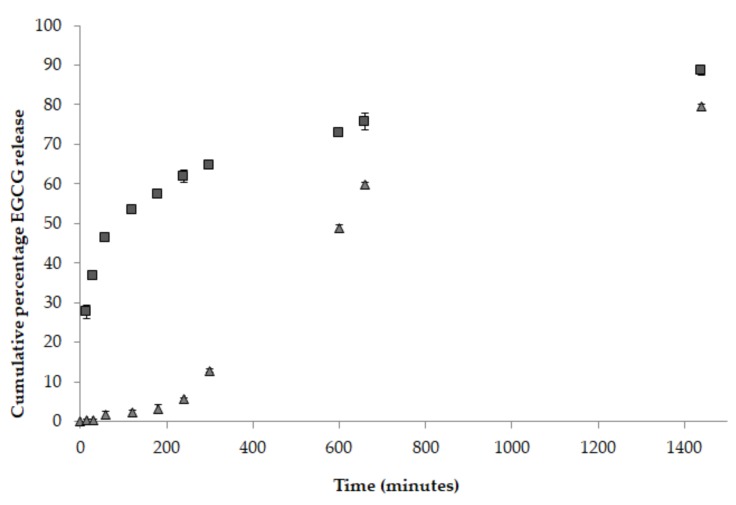
Cumulative percentage of epigallocatechin gallate (EGCG) release from non-coated microspheres (Ch-PP60_8:1_, ■) and Eudragit S-100 coated microspheres (S100Ch-PP60, ▲) in simulated gastrointestinal conditions. Error bars ± standard deviation (SD); *n* = 3.

Both Ch-PP60_8:1_ and S100Ch-PP60 were tested for their release profile in simulated gastrointestinal fluids using USP dissolution test apparatus at 37 ± 0.5 °C (Figure 1). The release profile of epigallocatechin gallate (EGCG) from non-coated microspheres (Ch-PP60_8:1_) and Eudragit S-100 coated microspheres (S100Ch-PP60) formulations were compared over the pH range from 1.2 (simulated gastric fluid) in acid buffer solution for 2 h, to pH 4.5 (simulated duodenum) for another 2 h, to pH 7.4 (simulated distal ileum and colon) for the remaining 20 h.

**Figure 2:**
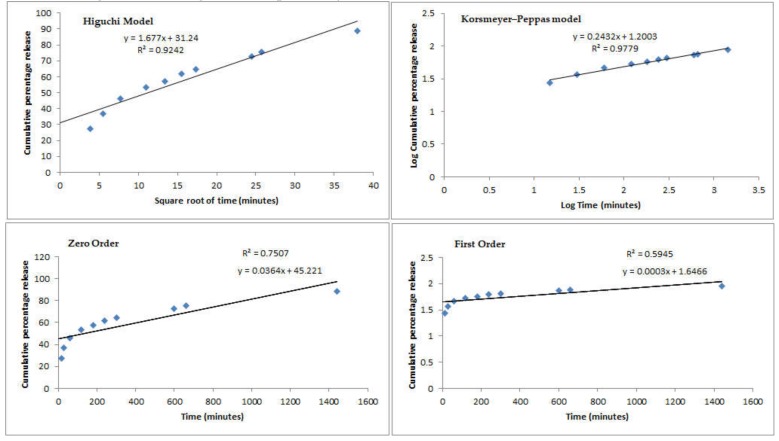
Mathematical fitting models (Higuchi model, Korsmeyer–Peppas model, zero order, and first order) of the cumulative percentage of EGCG release from non-coated microspheres (Ch-PP60_8:1_) in simulated gastrointestinal conditions.

**Figure 3:**
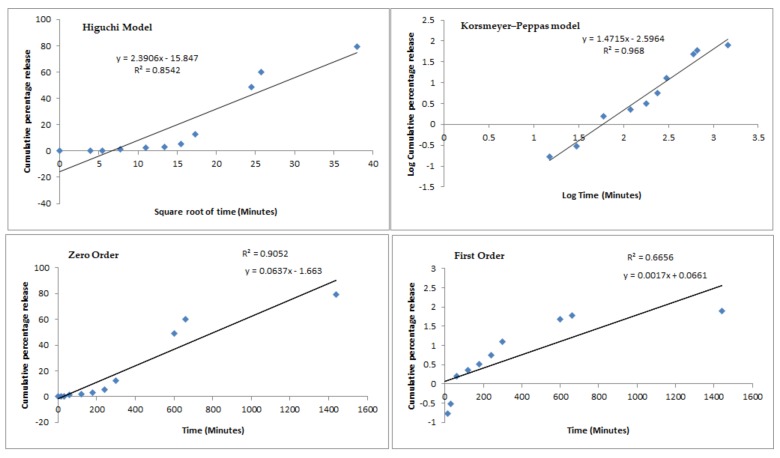
Mathematical fitting models (Higuchi model, Korsmeyer–Peppas model, zero order, and first order) of the cumulative percentage of EGCG release from Eudragit S-100 coated microspheres (S100Ch-PP60) in simulated gastrointestinal conditions.

Mathematical fitting models (Higuchi model, Korsmeyer–Peppas model, zero order, and first order) have been used to describe the recorded profiles from both tested batches and results are shown in Figure 2 and Figure 3, respectively.

The capacity of S100Ch-PP60 to neutralize reactive oxygen species (ROS) was evaluated using the DPPH scavenging assay, which was shown to be concentration dependent. The absorbance decay of the control test was compared with the recorded absorbance decay of Ch-PP60_8:1_ versus S100Ch-PP60, resulting in the percentage scavenging of free radicals translated as the antioxidant activity (Table 3) [33]. Differences were shown to be statistically significant. For the positive control (BHT) 78.11% scavenging of DPPH radical was recorded at the highest tested concentration (6.0 µg/mL) [33,34]. For the Ch-PP60_8:1_, the linear regression of *R*^2^ = 0.9941 (y = 4.2743x – 1.45) was obtained and the IC_50_ calculated as 212.3 µg/mL, which confirms the cross-linking of chitosan with glutaraldehyde did not compromise the antioxidant activity of catechin. The coating with Eudragit (S100Ch-PP60) resulted in the linear regression of *R*^2^ = 0.9895 (y = 3.1023x – 0.728) with the IC_50_ of 154.4 µg/mL.

**Figure 4:**
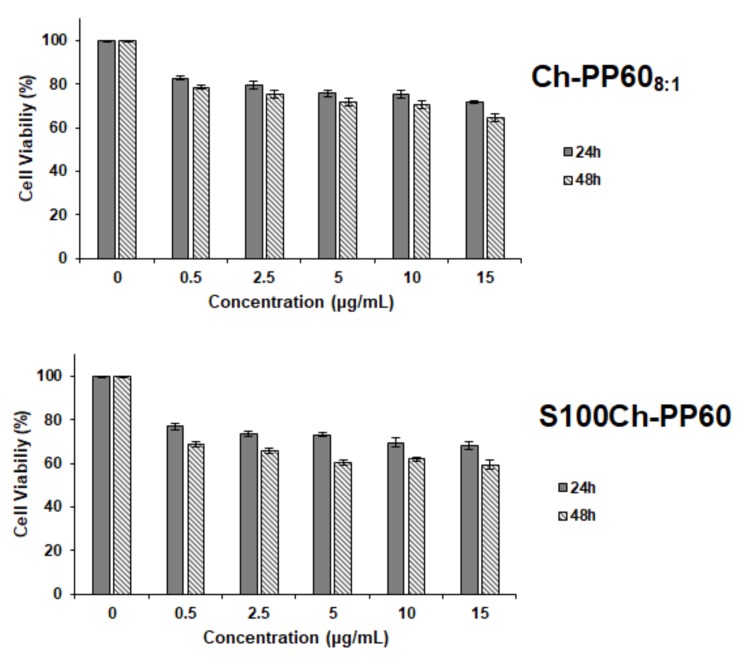
Evaluation of the cytotoxic activity of Ch-PP60_8:1_ and S100Ch-PP60 in Caco-2 cell line using the MTT assay at 24 and 48 h. The data represent the mean values ± SD (*n* = 3).

From the MTT assay (Figure 4), we can see that there was no significant difference in cell viability, over the concentration range tested, i.e., between 0.5 and 15 µg/mL. Despite the statistical (*p* < 0.05) significant reduction in cell viability observed in all tested concentrations and at both time-points (compared to the control), the cell viability remained above 70% at all tested concentrations for the non-coated microspheres, indicating limited risk of cytotoxic events. The Eudragit coating slightly reduced the cell viability. At the end of the 48 h, the reduction in cell viability was 35.41% and 40.63% for Ch-PP60_8:1_ and S100Ch-PP60, respectively, at the highest tested concentration.

## 4. Discussion

When varying the chitosan:PP60 ratio from 2:1 to 10:1, the mean diameter of microspheres increased from 5.57 μm to 7.83 μm (Table 2), which was an expected result as the increase of polymer concentration contributes to an increase the mean particle size. The higher encapsulation efficiency was obtained for the ratio 8:1 (Ch-PP60_8:1_, 87.21 ± 0.33%) with a loading capacity of 7.72 ± 0.11%. Increasing the amount of chitosan also resulted in the increase of the yield of production up to 89.99 ± 0.70%. When increasing the speed rotation from 1000 rpm to 2000 rpm, the size decreased from 9.22 μm down to 6.97 μm. This is attributed to the improved distribution of small emulsion droplets within the aqueous phase, which are then stabilized with the surfactant molecules. The highest yield of production (92.27 ± 0.55%), loading capacity (11.32 ± 0.41%), and encapsulation efficiency (83.55 ± 0.81%) were achieved with 1% (*m*/*v*) of surfactant concentration, with this amount suitable to cover all new particle surfaces being formed upon emulsion cross-linking. When varying the speed, no significant changes were seen for the loading capacity as the chitosan:PP60 ratio remained 4:1 (Table 1). The best results (highest YP%, LC%, and EE%) were obtained with the 1.0% (*w*/*v*) of Span 80, resulting in microspheres with a mean diameter of 6.45 μm. The formulations produced with a chitosan:PP60 ratio of 8:1 (Ch-PP60_8:1_) dispersed in 1% (*m*/*v*) Span 80 at 1500 rpm have been selected for further studies. The obtained microspheres (Ch-PP60_8:1_) were coated with Eudragit S-100 (S100Ch-PP60) and showed a significant increase of the *D_mean_* (51.4 µm). Eudragit S-100 is an anionic copolymer based on methacrylic acid and methyl methacrylate, both polymers contributing for the increase of the particles diameter which demonstrate that particles are coated with the enteric copolymer.

When comparing the release profile between Ch-PP60_8:1_ and S100Ch-PP60 in simulated gastrointestinal conditions, the results depicted a delayed release of the active ingredient when coating the chitosan microspheres with the enteric polymer. Within the first 2 h, about 53.45 ± 0.28% of EGCG released from non-coated microspheres was quantified in the dissolution medium, whereas only ca. 2.24 ± 0.52% was released from the coated microspheres. The increase of the pH to 4.5 induced the further release up to 61.90 ± 1.59% and 5.51 ± 0.22% by the end of the 4th hour from non-coated and Eudragit S-100 coated microspheres, respectively. After 24 h of assay, the cumulative amount reached 88.56 ± 1.24% and 79.54 ± 0.52% when released from non-coated and Eudragit S-100 coated microspheres, respectively. These results also demonstrate that S100Ch-PP60 was effectively coated with polyacrylic polymer and this is able to ensure an enteric resistance of the microspheres until they reach the colon. 

Comparing the *R^2^* values recorded for the different fitting models, the release plots of both profiles followed the Korsmeyer–Peppas model with the highest correlation coefficient values of 0.9779 (Ch-PP60_8:1_) and 0.9680 (S100Ch-PP60). When coating the microspheres with Eudragit S-100, the release mechanism fitted to the Power Law profile which follows the equation $M_{t}/M_{\infty }=k^{\prime }t^{n}$, where $M_{t}$ is the cumulative amount of active released at time t, $M_{\infty }$ is the cumulative amount of active released at infinite time, $k^{\prime }$ is the Korsmeyers–Peppas constant that is governed by the physicochemical properties of the microspheres. The diffusional exponent *n* translates the release mechanism, i.e., if *n* = 0.5 the release follows the Fickian diffusion, and if 0.5 < *n* < 1.0 it follows a non-Fickian diffusion. A Case II transport is seen as *n* approaches 1.0 when the release independent of time and reaches zero-order release; a super Case II transport is followed when *n* > 1.0 [35]. Interestingly the Eudragit coating significantly changed the transport mechanism; for the non-coated microspheres the release followed the Fickian diffusion, which means that the active was released from the glutaraldehyde cross-linked chitosan microspheres by the usual molecular diffusion attributed to a chemical potential gradient. With the enteric coating, a super Case II was approached, in which the transport mechanism of the active from the microspheres is associated with the erosion of polymeric coating, as seen with the increase of the pH up to 7.4 (Figure 1). The second-best fitting model for the coated microspheres was the zero-order release. The obtained profiles seem to be appropriate to the proposed colonic delivery of PP60. Indeed, it is expected that the release of the active is kept at minimum through the transport of the microspheres through the stomach and small intestine before they reach colon. Eudragit S-100 is soluble at pH above 7; when reaching pH 7.4 the amount of active being released increased almost 40%. The presence of a modified release profile in both formulations could be confirmed, with a drug protective effect promoted by the enteric coating.

The antioxidant activity represents the first step for the evaluation of health benefits [36,37,38]. For an effective activity against metabolic diseases, the well-known antioxidant activity of green tea should be kept until it is released from the microspheres. The DPPH test demonstrated that the scavenging capacity of S100Ch-PP60 was dependent on the concentration, i.e., the higher the concentration the higher the scavenging activity. The coating of the microspheres with the polyacrylic polymer did not compromise the antioxidant activity of the loaded PP60, a property that can be further exploited for the treatment/prophylaxis of metabolic diseases. 

Prior to any in vivo experiment, toxicological studies should be first performed in vitro, using cell models that mimic the body conditions, in order to minimize the number of animal studies and to have an idea of the cytotoxicity of the drug delivery system at an early stage. Although known to be biocompatible and biodegradable, glutaraldehyde-cross-linked chitosan microspheres should be characterized for their capacity to maintain the viability of cells in vitro. For oral delivery, the main barrier of drug absorption is the intestinal epithelium. The Caco-2 cell line is a human colon epithelial cancer cell line often used as a model to mimic the gastrointestinal conditions. While other cell lines are available for cyto/geno-toxicity assessment [39,40], this model seems to be the most realistic cell culture to test oral drug delivery systems. Cytotoxicity of drug delivery systems in the gut is frequently estimated by colorimetric methods in Caco-2 cells [27,41,42]. Among these methods, the most frequently used is the MTT assay. In the MTT assay, the mitochondrial function of the cells is tested. Only live cells will produce the enzymes capable to reduce the MTT reagent. The cytotoxicity of Ch-PP60_8:1_ was checked in the Caco-2 cell line, in comparison to the Eudragit-coated microspheres (S100Ch-PP60). From the obtained results, cells remained viable when treated with both Ch-PP60_8:1_ and S100Ch-PP60 over the tested concentration range over a period of 24 h or 48 h. The coating of the chitosan microspheres with the acrylic polymer reduced the cell viability down to approximately 60% at the highest tested concentration (15 µg/mL) after 48 h. The effect of size and concentration of particles on cell viability is well-described in scientific literature, being also very much dependent on the type of cell lines [43]. Monolayer type adherent cells, i.e., cells that adhere onto the surface of the culture dish as happens with Caco-2, are more sensitive to the effect of size and concentration as more surface area is exposed. The smaller the size and the lower the concentration, the higher the cell uptake which in principle would induce higher cytotoxicity [44]. Our results show that cell viability was slightly compromised by the coating with the polyacrylic polymer attributed to the density of particles onto the surface of the cell’s monolayer.

It is expected that S100Ch-PP60 microspheres can be further processed in foodstuff and in beverages to provide an alternative approach for the administration and delivery of phytochemicals with nutraceutical value. Indeed, micro/nano-nutraceuticals represent a useful tool in managing health conditions, particularly in patients not eligible for conventional therapy [45,46]. Follow up studies, use, and compliance [47,48,49,50], as well as communication strategies and assessment [51], should be applied also to nutraceuticals. This approach will allow the management of different health conditions, as happens with the metabolic syndrome [52,53], obesity, and dysmetabolism [54,55,56,57,58], which are often related to the food intake/dietary habits. Given its high levels of antioxidants and polyphenols, green tea is sometimes seen as the healthiest beverage on earth. It has recognized health benefits in metabolic syndrome, e.g., against fat gain, in preventing and managing type 2 diabetes, besides lowering the risk of cancer among others biological effects. Metabolic syndrome is a combination of risk factors ending up in chronic diseases, such as obesity, and is intimately related to oxidative stress and inflammation. As a prophylactic measure, antioxidants, such as those of green tea, can further be exploited in foodstuff as nutraceutical. The smart delivery of nutraceuticals [3,5,6,59,60,61,62,63,64,65], through their encapsulation in micro/nanoparticles, can offer an approach to increase their bioavailability. Besides, chitosan microspheres coated with an enteric polymer can be formulated in different food matrices for a modified release in the gut, offering the opportunity to enrich the nutraceutical value of food, supplements, and beverages recommended for the prevention and/or treatment of health conditions linked to dysmetabolism. Such micro/nano-based products should become part of an improved lifestyle as a prophylactic approach against metabolic disorders.

## 5. Conclusions

Polyphenon-60-loaded chitosan microspheres cross-linked with glutaraldehyde were successfully prepared. The microspheres were then coated with Eudragit and tested for their modified release in simulated gastrointestinal conditions. The delayed release of green tea was confirmed; both non-coated and Eudragit coated microspheres followed the Korsmeyers–Peppas release model, demonstrating capacity to retain the antioxidant activity of the active ingredient. The coated microspheres increased their size significantly, however without significantly compromising their biocompatibility with the intestinal epithelial Caco-2 cells. The potential for this formulation to deliver poorly water-soluble drugs, such as catechins, was illustrated and can be exploited for the management of metabolic diseases, exploiting the biological effects of green tea, as well as being applied to other matrices of vegetal origin and mucoadhesive chitosan microspheres. 

## Figures and Tables

**Table 1:** Variable parameters of chitosan microspheres produced by emulsion cross-linking method (PP60, polyphenon 60; rpm, rotations per minute; % (*w*/*v*), percentage weight per volume).

Constant Parameters	Processing Variables	Formulation Code
	Chitosan:PP60 ratio	
Rotational speed: 1500 rpm Concentration of Span 80: 1%	2:1	Ch-PP60_2:1_
	4:1	Ch-PP60_4:1_
	8:1	Ch-PP60_8:1_
	10:1	Ch-PP60_10:1_
	Rotational speed	
: Chitosan:PP60 ratio: 4:1 Concentration of Span 80: 1%	1000 rpm	*Speed*_10_
	1500 rpm	*Speed*_15_
	2000 rpm	*Speed*_20_
	Span 80	
Chitosan:PP60 ratio: 4:1 Rotational speed: 1500 rpm	0.5%	S80_0.5_
	1.0%	S80_1.0_
	1.5%	S80_1.5_

**Table 2:** Particle size, percentage yield, percent drug content, and entrapment efficiency of uncoated and Eudragit coated chitosan microspheres. The results were subjected to one-way ANOVA (Tukeys Test. Data are presented as mean ± SD (standard deviation); *n* = 3.

Formulation code	*D_mean_* (µm)	YP% (%)	LC (%)	EE (%)
Ch-PP60_2:1_	5.57	69.73 ± 0.27	18.36 ± 0.71	75.26 ± 0.27
Ch-PP60_4:1_	6.23	78.64 ± 0.76	13.91 ± 0.22	76.81 ± 0.55
Ch-PP60_8:1_	7.16	86.15 ± 0.88	7.72 ± 0.11	87.21 ± 0.33
Ch-PP60_10:1_	7.83	89.99 ± 0.70	6.99 ± 0.53	85.61 ± 0.14
*Speed*_10_	9.22	89.27 ± 0.45	12.91 ± 0.27	77.82 ± 0.91
*Speed*_15_	7.68	91.25 ± 0.34	13.84 ± 0.61	79.33 ± 0.17
*Speed*_20_	6.97	90.11 ± 0.56	13.25 ± 0.12	76.16 ± 0.73
S80_0.5_	11.82	88.37± 0.66	9.36 ± 0.11	81.11 ± 0.49
S80_1.0_	6.45	92.27 ±0.55	11.32 ± 0.41	83.55 ± 0.81
S80_1.5_	6.11	90.28± 0.47	10.51 ± 0.27	82.24 ± 0.77

**Table 3:** Percentage of scavenging of free radical DPPH (antioxidant activity; *AA (%)*) by Ch-PP60_8:1_ and S100Ch-PP60. Values are mean ± SD (*n* = 3).

µg/mL	AA(%)	
	Ch-PP60_8:1_	S100Ch-PP60
1	2.23 ± 0.98	1.83 ± 1.10
2	7.15 ± 0.03	5.24 ± 1.04
3	12.38 ± 1.04	9.44 ± 0.23
4	15.87 ± 1.02	12.29 ± 1.67
5	19.22 ± 0.33	14.65 ± 0.91
10	24.21 ± 0.75	17.33 ± 0.82
